# Perspectives of primary care providers and endoscopists about current practices, facilitators and barriers for preparation and follow-up of colonoscopy procedures: a qualitative study

**DOI:** 10.1186/s12913-018-3567-y

**Published:** 2018-10-17

**Authors:** Gayle Restall, John R Walker, Celeste Waldman, Kathleen Zawaly, Valerie Michaud, Dana Moffat, Harminder Singh

**Affiliations:** 1 Department of Occupational Therapy, College of Rehabilitation Sciences, Rady Faculty of Health Sciences, University of Manitoba, R106 - 771 McDermot Avenue, Winnipeg, MB R3E 0T6 Canada; 2 Department of Clinical Health Psychology, Rady Faculty of Health Sciences University of Manitoba, Winnipeg, MB Canada; 3 College of Nursing, Rady Faculty of Health Sciences, University of Manitoba, Winnipeg, MB Canada; 4 Interdisciplinary Studies, Faculty of Graduate Studies, University of Manitoba, Winnipeg, MB Canada; 5 Department of Internal Medicine, College of Medicine, Rady Faculty of Health Sciences, University of Manitoba, Winnipeg, MB Canada

**Keywords:** Information, Knowledge translation, Bowel preparation, Lower gastrointestinal endoscopy

## Abstract

**Background:**

Colonoscopy has become a common medical procedure due to increased use of colonoscopy for evaluation of symptoms, colorectal cancer screening and surveillance of people with higher risks of developing colorectal cancer. Timely access to colonoscopy is essential for diagnosis of colorectal cancer, as well as diagnosis and management of inflammatory bowel disease and gastrointestinal symptoms such as diarrhea. The purpose of this study was to obtain the perspectives of primary care providers and endoscopists about current practices, barriers and facilitators to following recommended practice for preparation and follow-up after colonoscopy. We also aimed to obtain recommendations for approaches to improve the process.

**Methods:**

Six focus groups (two with gastroenterologists, two with surgeons who perform colonoscopies and two with primary care providers) were held between October 2015 and January 2016. Analysis was performed using inductive qualitative approaches.

**Results:**

Variations and challenges in communication for continuity of care and understanding the distribution of responsibility were identified, as were perceived benefits and challenges of a central intake system for colonoscopies. Recommendations were made to improve processes including strengthening communication and information sharing. A comprehensive quality improvement plan would facilitate implementation of recommendations.

**Conclusions:**

Findings emphasize the need for improved patient-focused information resources for each step of the colonoscopy process and improved communication among practitioners. The findings apply to other services requiring collaboration among patients, primary care providers, and medical specialists.

**Electronic supplementary material:**

The online version of this article (10.1186/s12913-018-3567-y) contains supplementary material, which is available to authorized users.

## Background

Colonoscopy has become a common medical procedure due to increased use of colonoscopy for evaluation of symptoms, colorectal cancer (CRC) screening and surveillance of people with higher risks of developing CRC [1, 2]. Since most CRCs are diagnosed on colonoscopy, to ensure timely diagnosis of CRC, timely access to colonoscopy is essential. Similarly timely colonoscopy is critical for diagnosis and management of inflammatory bowel disease and gastrointestinal symptoms such as diarrhea [3–5].

One approach to increasing access to colonoscopy is to increase the number of colonoscopy procedures, available at any time, to reduce problems with waiting lists. This approach is costly in terms of health care resources required to increase the capacity to perform more colonoscopies. Another important approach is to make the most effective and efficient use of existing resources, including reducing the need to do early repeat colonoscopy. Bowel cleansing for colonoscopies has been reported to be poor in up to 20–40% of cases [6]. Poor bowel cleansing leads to repeat colonoscopies at short intervals, increased risk of complications, longer procedure times, and increased rates of missed lesions, including CRCs and CRC precursor colorectal polyps.

Adhering to recommended follow-up intervals can also contribute to the effectiveness and efficiency of the system. Many studies, including from Manitoba, Canada where this study was conducted, have documented follow-up colonoscopies at shorter or longer than the recommended time intervals [7–13]. Patients at higher risk of CRC may not receive appropriate follow-up, and for those with low risk, early follow-up colonoscopy increases the demand on scarce endoscopy resources and exposure to the risks associated with the procedure.

Another approach to increasing the efficiency of the system is for primary care providers (PCPs) to have a patient see the endoscopist the same day as the procedure without prior consultation (this is referred to as “direct to scope”, or “open access endoscopy”). Studies have suggested that patients referred direct to scope have shorter wait times [14]. Importantly, there is no documented difference between pre-procedure consultation and “direct to scope” approaches in the procedure-related anxiety of patients [15]. Although there are no guidelines and individual practices vary, common indications for direct to scope include screening colonoscopy, colonoscopy to follow-up other colon cancer screening tests such as a positive fecal occult blood test, surveillance colonoscopy and evaluation of iron deficiency anemia and rectal bleeding. However, anecdotally some endoscopists do not see patients direct to scope, but meet them ahead of time of colonoscopy and discuss the symptoms, indications and procedures individually with each patient themselves, rather than relying on discussion with the referring provider.

Ideally, colonoscopy should be performed for recommended indications, at recommended intervals, and when patients are aware of the reasons for colonoscopy, know the associated risks, understand and are well-prepared for the procedure, and have appropriate follow-up. To achieve that aim effective communication between PCPs, endoscopists and patients is essential.

## Methods

This study was part of a larger project investigating processes to improve the appropriateness and efficiency of colonoscopy procedures. Planning for the project was guided by Professional and Patient Advisory Committees. The overall purpose was to develop colonoscopy services that:

1. Reduce wait times for colonoscopy by eliminating the requirement for a pre-colonoscopy appointment with the endoscopist through “open access” or “direct to scope” processes, when appropriate
2. Enhance bowel preparation quality for colonoscopy
3. Ensure the appropriate interval for surveillance colonoscopy

The study was approved by the Health Research Ethics Board at the University of Manitoba.

### Objective

The purpose of this study was to obtain the perspectives of PCPs and endoscopists (gastroenterologists and surgeons) about current practices, barriers and facilitators to following recommended practice for preparation and follow-up after colonoscopy. We also aimed to obtain recommendations about approaches to improve the processes.

### Design

We used a qualitative research design following an interpretive description approach [16]. In this design nalysis evolves beyond pure description of a phenomenon to broader interpretations that have practical application.

### Procedures

Participants included PCPs, gastroenterologists and surgeons who were informed, in advance by email, about the focus group as the main topic of a regularly scheduled meeting. Only those who were willing to join in the focus group attended the meeting. Informed consent to participate was confirmed with the completion of a consent form at the start of the meeting. Most of the clinicians provided services in facilities managed by the Winnipeg Regional Health Authority (WRHA) where two-thirds of the colonoscopies in the province are performed. The WRHA had just started a central intake process for endoscopic procedures, which was being implemented in different facilities in stages.

We held six focus groups in Winnipeg between October 2015 and January 2016: two with gastroenterologists, two with surgeons who perform colonoscopies and two with PCPs. One focus group included PCPs providing services in the regions of the province with smaller population centres (up to several hours drive away by car or air travel). Often patients had to travel to Winnipeg for colonoscopy services.

Each focus group was facilitated by two members of the research team (one with expertise in conducting focus groups, the other with expertise in providing endoscopy services) with support from an additional member who managed the audio recording equipment and maintained written notes. A semi-structured interview guide (Additional file 1) was used to facilitate discussion. Focus group recordings were transcribed verbatim and verified by a member of the research team.

### Data analysis

Inductive qualitative analysis was performed following methods described by Miles, Huberman & Saldana [17] using NVivo 11 software. CW read through all transcripts several times, then developed and documented an overall sense of the findings in relation to two analytical questions: What is going on (i.e. what are current practices/processes) and what is being learned (to improve the practices/processes)? Next, she developed a coding scheme based on line-by-line coding of the transcripts. A second member of the team (KZ) independently developed a coding scheme through line-by-line analysis of the transcripts and identified preliminary broad themes, which were used in subsequent analysis. These two coding schemes were compared by GR who merged, renamed, and refined codes through a second cycle of analysis to develop broader categories. Next, matrices were developed across classifications of participants (i.e., PCPs, surgeons and gastroenterologists). Broad themes and the interrelationships between themes were developed. Network relationships explored the interrelationships of processes. Interpretations were presented to two leaders of the research team who provided clinical and conceptual perspectives prior to member checking. Member checking was used to obtain stakeholder feedback about the findings. Member checking consisted of distributing a draft summary report to a subgroup of 34 stakeholders comprised of focus group participants who provided their e-mail addresses to the researchers and members of the professional advisory group for the overall project. Ten people responded. Comments were analyzed, coded for themes, and are summarized at the end of the results section.

## Results

We conducted six focus groups with 46 participants between October 8, 2015 and January 28, 2016. There were 4 to 11 participants in each focus group. PCPs (*n* = 16; 50% male) participated in one of two focus groups; gastroenterologists (*n* = 15 including 4 residents; 100% male) in another one of two groups, and surgeons (*n* = 15; 86.7% male) in the remaining two focus groups. The participating gastroenterologists and surgeons were all endoscopists and, hence, when referring to both the groups together, we refer to “endoscopists.” PCPs had less than 1 to 33 years of practice (*n* = 14; median = 17) and endoscopists, not including residents, had 2 to 38 years of practice (*n* = 21; median = 15). Data for years of practice were missing for two PCPs and five endoscopists.

Participants asserted that continuity of care across PCPs and endoscopists was very important to provide high quality care to patients. The complexity of the system, particularly regarding patterns of communication, and collaboration through the distribution of responsibility among the patient, PCP, and endoscopist could work well in many instances but also presented numerous challenges for promoting safe and efficient referral and follow-up. A central intake service that included the potential for an increase in patients attending “direct to scope” could streamline services and, at the same time, create new challenges for communication, understanding of distribution of responsibility, and, ultimately, the efficiency and quality of care.

The findings from this study are reported below in themes of a) communication for continuity of care b) understanding the distribution of responsibility and c) the perceived benefits and challenges of a central intake system for colonoscopies. When differences among the three types of provider were evident in the matrix analysis, we report the ways in which perceptions differed.

### Communication for continuity of care

**Fig. 1:**
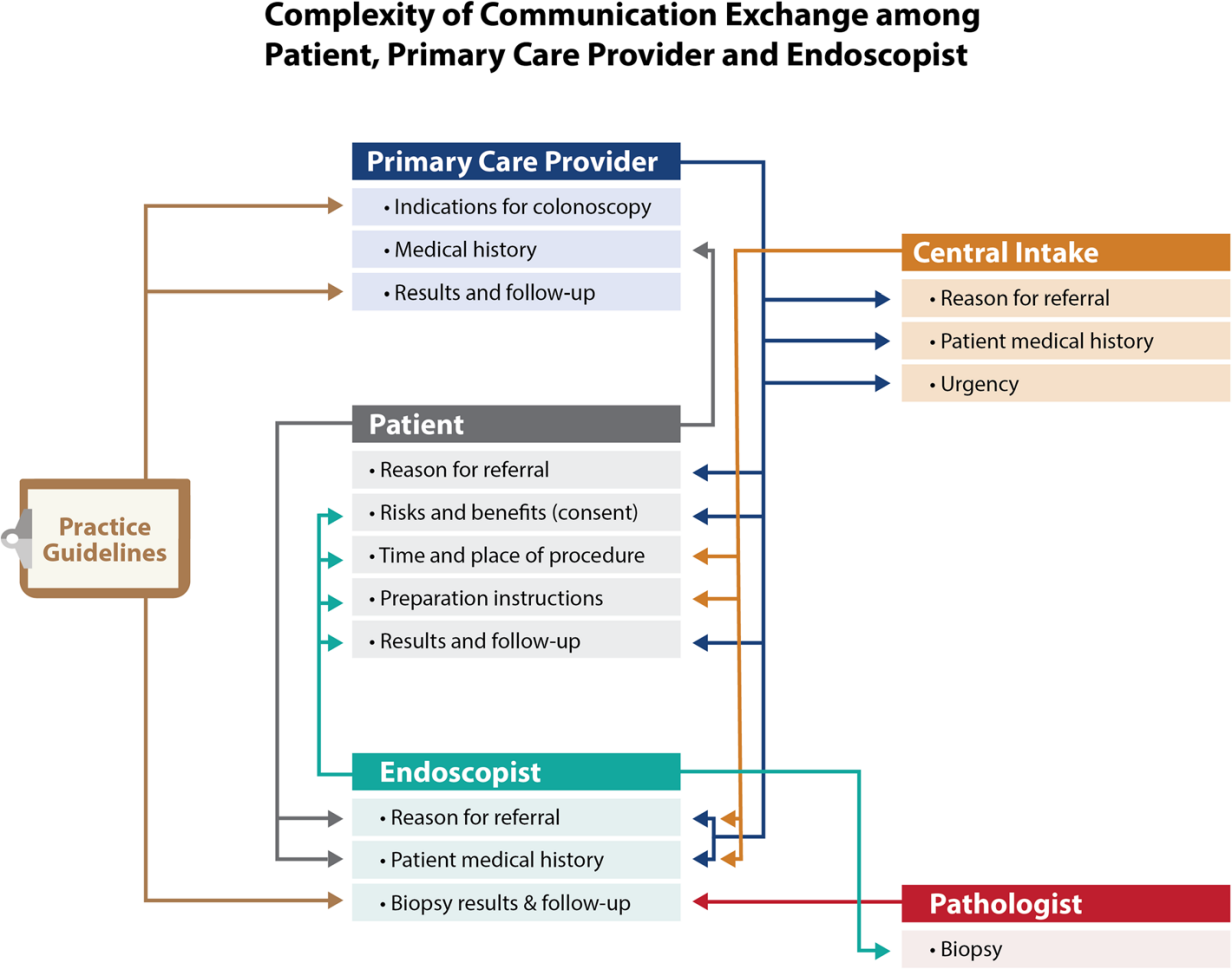
Communication for collaboration among the patient, primary care provider and endoscopist for preparation and follow-up of colonoscopy procedures. Appropriate and quality colonoscopy procedures and follow-up require a consistent, accurate and timey flow of information among the patient, primary care provider and endoscopist

Figure 1 illustrates the information that each group requires and the potential lines of communication for information. Participants in the focus groups described these lines of communication along with inconsistencies in communication processes across practitioners. Communication challenges included insufficient information, inconsistent information, difficulties in interpreting important information, and breakdowns in the communication channels. These challenges were evident both pre- and post-colonoscopy.

#### Pre-procedure communication

Pre-procedure communication from the PCPs could go directly to the endoscopist. However, with the development of a central intake system, the information was increasingly passed through that system, creating an intermediary step along the path.

##### Endoscopists’ perspectives

The endoscopist groups discussed inconsistencies in the amount and type of information they received from PCPs at the time of referral. One surgeon noted, “Most (of my consults) have appropriate amounts of information on them. But it really depends on whoever the referring doctor is and how much interest they have in that particular disease process.” The challenge related to these inconsistencies was summarized in one surgeon focus group as, “we don’t have a standard of information that should be present on all (patients) prior to the… consult being sent.” Both surgeons and gastroenterologists noted that referrals often lacked details of medical and family history, and blood work reports. This was problematic particularly in some direct to scope situations when the endoscopist considered that there was insufficient information to ensure a safe procedure or to limit unnecessary procedures. Therefore, many endoscopists preferred to see the patient prior to the procedure to obtain necessary information. One gastroenterologist said, “I wouldn’t feel comfortable with (a patient) who I’ve never met because… a good percentage of the time, (patients) don’t need another colonoscopy… They have already had it done. It’s just not put in their referral history.” Another noted that he sometimes will “get a sense (from the referral that the) patient doesn’t need a scope” but will make that determination after seeing the patient himself. Another said, “I have this concern that many people, a large number of people, are getting colonoscopies unnecessarily, and the main reason is that no one’s ever sat down and talked to them.”

One endoscopist noted that “a lot of the family physicians are doing an excellent job educating their patients,” but went on to say that some patients arrived with insufficient information about why they were being referred for a colonoscopy. In other cases, insufficient patient understanding of the reason for the colonoscopy could result in patients not arriving for the procedure after being scheduled. Some endoscopists reported that a pre-clinic appointment was helpful for sharing information about the procedure, particularly with patients who had little knowledge about the purpose or process. This conversation also could allow patients to decline the procedure once they learned more about the procedure and potential associated benefits and risks. One surgeon said, “I also talk a lot to my patients about the risks and benefits (of the colonoscopy), and I would say, not uncommonly, I have patients decline a colonoscopy right there.” In contrast, some endoscopists reported that some patients were very well informed about the procedure and viewed the pre-procedure appointment as a waste of time. This was also true of people who were worried about a potential cancer diagnosis. One surgeon said, “There are patients who worry about cancer, (instead of waiting) to be seen by somebody to explain it, (they prefer) going directly for their scope and getting it done as quickly as possible and getting their problem dealt with.”

Obtaining consent for the procedure was an important topic of discussion among focus group participants. Endoscopists thought it was important for patients to have information about the risks and benefits in advance of direct to scope procedures. They expressed concerns about getting consent on the day of the procedure, believing that it was difficult for a patient to decline once they had prepared for colonoscopy (including taking the required laxative) and come to the facility. One surgeon stated, “Are people going to say ‘no’ when they’ve done a full bowel prep? And certainly, at that point, people don’t.” Some felt that it was important for PCPs to communicate the information supporting an informed decision (benefits and risks) in advance, to provide patients the information and time to decide whether they wanted the procedure. One endoscopist talked about the process of sending a consent-related brochure to the patient in advance of the procedure and then being available to answer the patient’s or family member’s questions.

##### PCPs’ perspectives

In the focus groups, PCPs discussed their responsibility to inform patients about the reasons for the procedure and some acknowledged their responsibility to obtain consent for the referral, including discussing the potential risks and benefits of a colonoscopy. Some reported feeling comfortable sharing this information with patients, as long as they had easy access to accurate and current resources. One PCP said, “I’m comfortable with (providing information about the procedure to patients). It certainly helps to have resources to do it…. if that pamphlet was right there and you just printed it off right in front of you. It would be designed perfectly… so that it ensures that I say the right things and I give (information to the patient about) the right complications.”

One of the areas in which PCPs did not feel comfortable in providing information to patients was related to bowel preparation. They noted that different endoscopists had different preferences for bowel preparation and that it was not helpful for the PCP to provide information that may contradict information received later from the endoscopist. One PCP said, “My experience is that there isn’t really consistency in that (bowel preparation instructions). It switches and I don’t know what it will be switched to.”

#### Post-procedure communication

There was consensus across all six focus groups that a major challenge was communication after the colonoscopy to ensure that PCPs and patients understood the results and that recommendations were followed. There was wide variation in the ways that endoscopists communicated findings and follow-up recommendations to patients and PCPs.

### Communication with patients

#### Endoscopists’ perspective

There was great diversity in approaches to communicating the results of the colonoscopy. Communication sometimes occurred verbally directly after the procedure between the endoscopist and the patient, the person accompanying them, or both. With the exception of times when the endoscopist felt that an immediate referral to, for example, an oncologist, was necessary, there was a widespread view that verbal discussion with the patient immediately after the procedure was ineffective. Most patients would be under the influence of the sedating medications which may impair memory. Some endoscopists reported that they provided patients a written summary of the findings that could include photographs of findings observed during colonoscopy. These reports were generated in facilities with the necessary computer software. In other cases, when the appropriate technology was not available, endoscopists provided hand-written notes. Some endoscopists called the patient back for a follow-up visit although this was more frequently done when there were pathological findings. However, one endoscopist who reported he saw “more patients in follow-up than maybe some other endoscopists,” indicated “I haven’t found it reliable over the years to trust the (PCP) to relate the specialist’s information and implication of (the findings).” This person also believed that follow-up with the endoscopist was “more satisfying for the patient.” Communication with patients requiring language interpretation presented additional challenges. One surgeon noted the need to have an interpreter available if there was a cancer diagnosis. He said, “I just don’t want to be going through the family (for discussing the presence of cancer).”

An area of concern was whether patients would remember when they should return for follow-up, particularly when the recommended follow-up colonoscopy was several years later. One surgeon indicated that it was important for patients to keep track of follow-up times in addition to having a flag in the medical record and informing the PCP. He said, “And I usually say, (to the patient), five years goes by a lot faster than you think, so what you should really do is get your calendar out and mark about four years on the calendar. And when four years rolls around, call my office again and we’ll get you back in to see me.”

#### PCPs’ perspectives

PCPs noted that patients sometimes turned to them with questions about the recommendations from the endoscopists. PCPs appreciated having the results before the patient returned to their office. Having timely and clear results and recommendations helped PCPs to interpret findings and reinforce recommendations. However, as noted in the next section, there were inconsistencies in the type and quality of information provided to PCPs.

### Communication between the Endoscopist and PCP

There was consensus among all groups that it was important for the PCP to receive information about the results of the colonoscopy, along with recommendations for follow-up. However, as one endoscopist noted, “The big issue is communication after the scope.” Similar to communication with patients, there was great diversity of endoscopists’ approaches to this communication. The timeliness of the communication varied. One PCP noted that sometimes “I get a report back within a day or two of the colonoscopy or the day of the colonoscopy and that’s fantastic; other specialists, it’s three to six month later.” Some PCPs noted that, at times, the report and recommendations from the endoscopist didn’t come at the same time as the pathology report, which created some confusion. One endoscopist addressed the uneven timing of when the colonoscopy report was sent and when the pathology report was returned by writing a note on the pathology report if the pathology findings suggested a different recommendation than the earlier post-procedure report. However, others reported they had to pull the chart or the colonoscopy report to recall the initial recommendation if it was not available with the pathology report. Another challenge for PCPs was the variability in the clarity of the results and recommendations. This led one PCP to send consults for colonoscopies to those specialists who wrote clear reports.

#### Clarifying responsibility

Participants noted that inconsistency of communication approaches meant that there was often lack of clarity concerning responsibility for arranging required follow-up after a colonoscopy. When the procedure indicated a high risk diagnosis, (e.g., cancer or Lynch syndrome), endoscopists in the focus group were most likely to take immediate action by calling the PCP, making a referral to an oncologist, and/or booking the patient for a follow-up appointment or colonoscopy. One endoscopist stated, “If I suspect that they have Lynch syndrome … as soon as they have (the colonoscopy) they are rebooked in a year for another colonoscopy. If they (have familial adenomatous polyposis), it’s the same.” This specialist felt that it was important for patients with these conditions to form a relationship with a consistent endoscopist and took responsibility for follow-up.

In contrast, the responsibility was more ambiguous when patients had colorectal polyps that were not likely to be cancerous, or had negative colonoscopy findings. In those cases, there were inconsistencies in approaches and participants voiced concern about two problems. First, some patients were being referred for colonoscopies too frequently. One endoscopist stated, “I get letters from docs saying, you know, this person has been getting scopes every year because they have polyps, and then I’ll go over their scopes and reports and realize they’re getting scoped way too often.” The other concern was for patients not having follow-up at intervals recommended by guidelines. Ensuring that the PCP had clear information from the endoscopist about the recommended follow-up was an approach used by some endoscopists. However, it was not always clear who would take responsibility for keeping track of and scheduling the recommended follow-up. One PCP stated, “There has to be a very clear understanding of whose role it is to do what.” In addition, participants acknowledged that some patients lived in communities in which there was a frequent turnover of PCPs and were concerned that a lengthy timeframe for the patient’s follow-up appointment would not be adequately monitored. Many endoscopists did not book follow-up colonoscopies and were concerned that patients would not be referred for the recommended follow-up. One approach by endoscopists focused on ensuring redundancy in communication of recommendations that could include a note on the medical record and informing both the PCP and the patient. One endoscopist said, “I think the more checks that you have in place, the less likely someone is going to fall through the cracks.”

### Benefits and risks of a central intake system for colonoscopy services

At the time of the study, a central intake (CI) system was being implemented in the region on a stepwise basis. Some participants had experience with CI while others did not. However, many participants had opinions about the risks and benefits of the system.

Concerns from PCPs included not being able to choose to refer to specific endoscopists whom they felt met a high standard. One PCP said, “(I need) to be comforted by knowing that, whoever is in charge of (central intake) knows each one of these endoscopists and would vouch for them, would be willing to have their own colonoscopy by each one of these people.”

PCPs were also concerned that CI would not allow them to identify patients who need more continuity or special attention from the endoscopist. This concern was shared by endoscopists who believed that lack of attention to engaging patients, or their support networks, could result in patients not arriving for the procedure or arriving with poor preparation. This issue was particularly salient for patients who had limited ability to speak or read English, who lived in remote communities, or who had limited access to a private toilet. One endoscopist commented, “I think central intake works very well for the population who has access to resources but for the people who don’t, it’s difficult.”

Participants also noted the benefits of CI in terms of efficiency as well as standardization of processes. A standardized referral form was viewed as a potential way to ensure that all PCPs provided the necessary referral information. Standardization of follow-up was also viewed as a benefit. One endoscopist said, “One of the biggest advantages of this central intake system would be the ability to standardize some of these things: a standardized follow-up.”

### Summary of member checking responses

Respondents agreed with the content of the focus group summary and most offered suggestions for improving the system. Several called for development of a comprehensive quality improvement plan that would identify priority issues and address them. Respondents identified several strategies for improving communication amongst the patient, PCP and endoscopist, with CI often having an intermediary role. Strategies included the development of brochure(s) provided to patients well in advance of the procedure, with easy to understand information about the procedure, the benefits and risks, and consent. Education of PCPs about colonoscopies with clear indicators for referral was also identified as an important strategy that could also include a feedback loop to PCPs to inform them about referrals that did not meet the criteria for colonoscopy. Several respondents also noted the importance of routinely including both the PCP and the patient in correspondence outlining follow-up plans and recommendations. One respondent advocated for the development of a “robust referral management system” that was accessible to patients to view. In the context of these suggested strategies, several respondents highlighted the need for flexibility in the system, in particular, for patients with low literacy, who live in rural or remote communities, or have limited resources.

## Discussion

As shown in Fig. 1, the communication required for colonoscopy is complex – involving the patient, the primary care provider, the endoscopist and the pathologist. Effective communication is essential at key times in the process such as referral, scheduling, bowel preparation, procedure day, and follow-up. Our findings suggest a large variability and complexity in the system of referral and follow-up for colonoscopy procedures and a desire among health care providers for improvements in the processes. The pre-procedure risks for patients associated with this variability focused on concerns about patients, PCPs and endoscopists getting adequate information in a clear and timely way to ensure a safe and effective procedure. Variability in practice also created risks that patients would not receive follow-up consistent with practice guidelines. Participants also noted that variability in practices created inefficiencies in the system through unnecessary procedures resulting in inflated and avoidable costs to the health care system and risks to patients.

Direct to scope procedures were acceptable to many endoscopists in our focus groups as long as PCPs provided them with sufficient information, clinical practice guidelines for referral for colonoscopy were being followed, and patients were well informed about the benefits and risks of the procedure. PCPs identified the need for accurate and current information at their fingertips to be able to identify the need for colonoscopy and to enhance their ability to provide the appropriate information to patients.

Direct to scope procedures are widely practiced. A survey of American Society of Gastrointestinal Endoscopy (ASGE) members in 1997 reported more than 60% of ASGE members were performing direct to scope procedures; a subsequent study reported a fivefold increase between 2000 and 2008 [18]. Direct to scope procedures are performed mostly for CRC screening and surveillance [19] and more often in non-hospital endoscopy units [20]. Several studies have documented direct to scope procedures can lead to shorter wait times, which is particularly important for diagnostic procedures when the risk of serious pathology is higher (for example, among elderly patients with persistent rectal bleeding or iron deficiency anemia [14]. Direct to scope procedures can also reduce the cost for patients and the health care system [18]. However, previous studies have also documented concerns such as endoscopies for unclear/not uniformly accepted indications [21], less properly informed patients [22], more missed appointments and last minute cancellations [19], important information missing in referral letters [23], worse bowel preparation quality and fewer significant findings on endoscopy [24]. It is concerning these issues remain prevalent even after decades of direct to scope practice. Improved communication at the point of referral is essential to improve the efficiency of direct to scope procedures and improve the functioning of the more traditional consultation route. As noted by our focus group participants, this would include standardized referral forms collecting adequate information and with predefined well-accepted indications for the direct to scope pathway (e.g. positive fecal occult blood test for CRC screening, unexplained iron deficiency anemia). Improved low cost educational materials for patients, available for distribution by PCPs would assist in informing patients about colonoscopy, its risks and benefits, and about the procedures required for bowel preparation. High quality educational materials would allow patients more time to consider the recommendation for colonoscopy and would make the process of obtaining informed consent easier and more consistent. A limited number of bowel preparation alternatives, details of which are readily available to the PCPs would make it feasible for PCPs to provide at least preliminary bowel preparation information to patients. The direct to scope pathway has many advantages and will continue; it is important to improve the process. A recent nationally representative PCP sample in the US reported that the majority of the pre-colonoscopy care was co-ordinated by endoscopists rather than PCPs [25]. This practice does not seem efficient. Consistent with the focus group findings, increasing capacity for PCPs to make appropriate direct to scope referrals is required and could include education of PCPs as well as improving communication between PCPs and endoscopists [26].

Methods to improve post colonoscopy information could include standardized written endoscopy and pathology reports provided to patients as well as the PCPs, developing standardized databases for patient/PCP reminder for follow-up and using patient-friendly educational materials describing common findings such as colorectal polyps. The wider use of electronic endoscopy reporting systems has made it more feasible to have standardized reports accepted by each service. Unfortunately, many practices do not use such systems. Centralized and standardized reminder systems for follow-up colonoscopy, would reduce the frequency of too early, too late or missed follow-up colonoscopies.

Centralized referral systems for colonoscopy are being used more widely based on the promise of a common queue leading to shorter wait times and more effective use of shared services. However, as highlighted by our focus group participants, it is important such systems have robust quality assessment and improvement initiatives to ensure that all patients, irrespective of the provider they see, get high quality care (before, during and after endoscopy). Previously, endoscopists, who PCPs perceived to provide better quality care, received more consultations. This may no longer be the case through a common queue system.

Focus group participants made clear recommendations for improvements in the consistency of communication among the primary care provider, the patient, and the endoscopist. A step-wise process for this communication is described in Table 1. Improved patient-focused information resources for each step of the colonoscopy process (referral, scheduling, bowel preparation, procedure day, and follow-up) would facilitate this communication.

**Table 1:** Recommendations for Communication

Patient as the focus of communication			
Time of referral - *Primary Care Provider*	Scheduling procedure - *Central Intake or consultant’s office*	Day of colonoscopy - *Endoscopist*	Post-procedure - *Endoscopist*
**Step 1**: Identify reason for referral and confirm reason is an indication for referral	**Step 1**: Schedule procedure or consultation appointment	**Step 1**: Review informed consent and answer any questions	**Step 1**: Review findings and determine patient follow-up for any clinically significant findings from pathology report (that will arrive later) Inform provider and patient of findings and recommendations
**Step 2**: Identify care pathway, i.e., direct to scope or consultation with the endoscopist	**Step 2**: Provide written information to patient about colonoscopy benefits and risks and specific bowel preparation Provide copy of consent form Provide supplemental web and/or video information if available	**Step 2**: Complete colonoscopy procedure	**Step 2**: Clarify responsibility for arranging recommended follow-up (including recommended repeat colonoscopy) – primary care provider, endoscopist, and /or patient.
**Step 3**: Review recommendation with patient including reason for referral and care pathway Provide written, patient-friendly information about colonoscopy, risks and benefits, and preparation for colonoscopy or about consultation with specialist	**Step 3**: Confirm appointment about one week in advance of date Provide information concerning cancellation and resources for questions about preparation and procedure as well as a reminder to patient to review information	**Step 3**: Provide written information about findings to patient and forward report to primary care provider; information includes comments about samples sent to pathology Inform patient and provider about recommended follow-up that may be modified based on any significant pathology findings Schedule follow-up appointment, if necessary	
**Step 4**: Obtain patient consent for referral		**Step 4**: Provide post-procedure instructions in writing to patient and person accompanying the patient	
**Step 5**: Complete standard referral form identifying priority or urgency Document any special patient-specific needs (e.g., travel, language, other assistance)			

The issues identified in this study of colonoscopy would apply to many other medical procedures. For these procedures, an assessment of typical approaches to communication among the patient, primary care provider, and specialist may assist efforts at quality improvement. The focus in our findings on the importance of clear communication with patients is consistent with the large body of work on shared decision making between patients and health care providers. Joseph-Williams and colleagues conducted a systematic review of patient-reported barriers and facilitators to shared decision making in health care [27]. They identified factors in both how the health care system is organized and in characteristics of interactions around health care decisions. Prominent factors included limited time in health care interactions and challenges in continuity of care among health care providers. Patients differ in their preference and ability to participate in health care decisions. Some have a view of the patient role as passive and consider that asking questions or requesting more information is disrespectful of the expertise of the health care provider or is inappropriate in some other way. Health care providers differ in their skill and knowledge around involving patients in decision-making.

Previous studies also suggest concern about the adequacy of procedures to obtain informed consent for medical procedures. In a systematic review of procedures to improve the process for obtaining informed consent for surgery and other invasive medical procedures, Kinnersley and colleagues considered some of factors that may make the process of obtaining informed consent difficult [28]. There is often time pressure in the situation and patients have little time to consider their options. The information can be quite technical and it may be difficult to communicate benefits and risks to patients in everyday language. Clinicians may have limited time to consider the patient’s concerns and the patient may not have the opportunity to express their concerns adequately. Patients may have limited time or confidence in seeking additional information. The use of a consent form may create a ritual in which there is limited exchange of information. Shared decision-making and informed consent are more likely to occur when there are quality educational materials in patient-friendly language available for patients and health care providers.

### Strengths and limitations of the study

The study took place in one jurisdiction and the experience may differ in other systems of care. However, the importance of clear lines of communication identified in this study are relevant to many jurisdictions and specialist procedures. This phase of the study did not directly obtain the perspectives of patients. However, in other phases we surveyed patients about their experiences with colonoscopy procedures [29] and completed individual patient interviews (manuscript in preparation). Focus group methodology uses the blended voice of the entire group so dissenting voices may not have shared opinions because participants may have been reluctant to disagree with their colleagues. However, our observation was that, overall, participants were willing to share openly, both positive and challenging experiences.

## Conclusion

Our focus groups identified communication challenges and potential solutions. Our regional health care system is in the process of implementing several of the recommendations. These recommendations may be helpful for other systems in their work on quality improvement.

## Additional file

Additional file 1: Semi-structured interview guide (DOCX 16 kb)

## Abbreviations

ASGE: American Society of Gastrointestinal Endoscopy
CI: central intake
CRC: colorectal cancer
PCP: primary care provider
WRHA: Winnipeg Regional Health Authority
