# Supplementary material for: Perspectives of primary care providers and endoscopists about current practices, facilitators and barriers for preparation and follow-up of colonoscopy procedures: a qualitative study
Source: BMC Health Serv Res. 2018 Oct 17;18:782. doi: 10.1186/s12913-018-3567-y (PMC6191911; doi:10.1186/s12913-018-3567-y)
Supplement: Supplementary file 1 — Semi-structured interview guide (DOCX 16 kb) [file 12913_2018_3567_MOESM1_ESM.docx]

Semi-structured Interview Guide

1) From your perspective and the perspective of the patient, how well do existing resources and procedures work in:

1. Referring the patient? What could be done to improve this aspect?
2. Communication between the family doctor and the endoscopist? What could be done to improve this aspect?
3. Communication between the family doctor and the patient? What could be done to improve this aspect?
4. Providing information on colonoscopy procedures and bowel preparation to the patient? What could be done to improve this aspect? Could the written information provided to the patient be improved? How?

For patients who are familiar with the internet, would it be helpful to have a location they could visit for information and to see a video about the patient experience during colonoscopy?

2) Research indicates that the use of split dose bowel preparation, where the patient receives half the dose the day before the colonoscopy and the rest early on the day of the colonoscopy, with colonoscopy starting within 4 hours of the end of the intake, results in fewer patients who have poor preparation and consequently less adequate colonoscopy. Are there impediments to use of split-dose bowel preparation? What could be done to improve this?

3) Some people advocate the use of a direct to scope approach where the family doctor is responsible for informing the patient about the colonoscopy and the preparation required. This would allow endoscopists to see more patients and see them more promptly.

1. From your view, what are the advantages and disadvantages of this approach?
2. Are there barriers to the use of direct to scope colonoscopy? What could be done to improve this?

4) Providing feedback on results of colonoscopy for colorectal cancer screening and recommendations for future follow up to the family doctor. What could be done to improve this aspect?

1. How familiar are you with current recommendations for follow-up after removal of polyps on colonoscopy and for patients with family history of colorectal cancer and polyps?
2. Would you be interested in using a downloadable app for physicians (and their smart phones) that would allow you to input information about the patient, their family history and the colonoscopy findings to find recommendations concerning follow up? Would you be more interested in a website which allows you to do this?
3. Would you prefer it if the endoscopist just provided recommendations for future follow up?

5) Providing feedback on results of colonoscopies and recommendations for future follow-up to the patient? What could be done to improve this aspect?

1. Would it be helpful to have standardized material in patient-friendly language that could be provided by the endoscopist and the family doctor?

6) Would it be helpful to have a standardized or centralized procedure for informing the patient and their physician about scheduling a follow up colonoscopy, close to the time a colonoscopy is recommended? This would require many resources and therefore may not feasible soon. Do you think the health authority should invest in such a system, if and when, resources can be allocated?

7) Are there any other suggestions for strengthening our systems?
